# β-Adrenergic stimulation recruits additional contracting sarcomeres in cardiomyocytes

**DOI:** 10.1093/cvr/cvag083

**Published:** 2026-04-24

**Authors:** Jia Li, Joakim Sundnes, Magnhild Sekse Erdal, Yufeng Hou, Per A Norseng, Wolfgang A Linke, Martin Laasmaa, William E Louch

**Affiliations:** Institute for Experimental Medical Research, Oslo University Hospital and University of Oslo, Ullevål Kirkeveien 166, 0450 Oslo, Norway; Simula Research Laboratory, Lysaker, Norway; Institute for Experimental Medical Research, Oslo University Hospital and University of Oslo, Ullevål Kirkeveien 166, 0450 Oslo, Norway; Institute for Experimental Medical Research, Oslo University Hospital and University of Oslo, Ullevål Kirkeveien 166, 0450 Oslo, Norway; Institute for Experimental Medical Research, Oslo University Hospital and University of Oslo, Ullevål Kirkeveien 166, 0450 Oslo, Norway; Institute of Physiology II, University of Münster, Münster, Germany; Institute for Experimental Medical Research, Oslo University Hospital and University of Oslo, Ullevål Kirkeveien 166, 0450 Oslo, Norway; Institute for Experimental Medical Research, Oslo University Hospital and University of Oslo, Ullevål Kirkeveien 166, 0450 Oslo, Norway; Simula Research Laboratory, Lysaker, Norway

**Keywords:** Cardiomyocyte, Sarcomere, β-Adrenergic stimulation, Calcium homeostasis

**Time of primary review: 20 days**

Traditionally, it was assumed that sarcomeres contract synchronously across the cardiomyocyte during the action potential. However, recent work has questioned this tenet.^1,2^ Using new methods to track individual sarcomeres, we found that ≈15–20% of sarcomeres activated at resting cell length exhibited stretch instead of shortening.^1^ When the cell was lengthened, these sarcomeres started to contract, supporting length-dependent activation. Thus, graded sarcomere recruitment may contribute to cardiac contractile reserve. We presently hypothesized that β-adrenergic stimulation might also recruit additional contracting sarcomeres to support inotropy.

**Figure 1:**
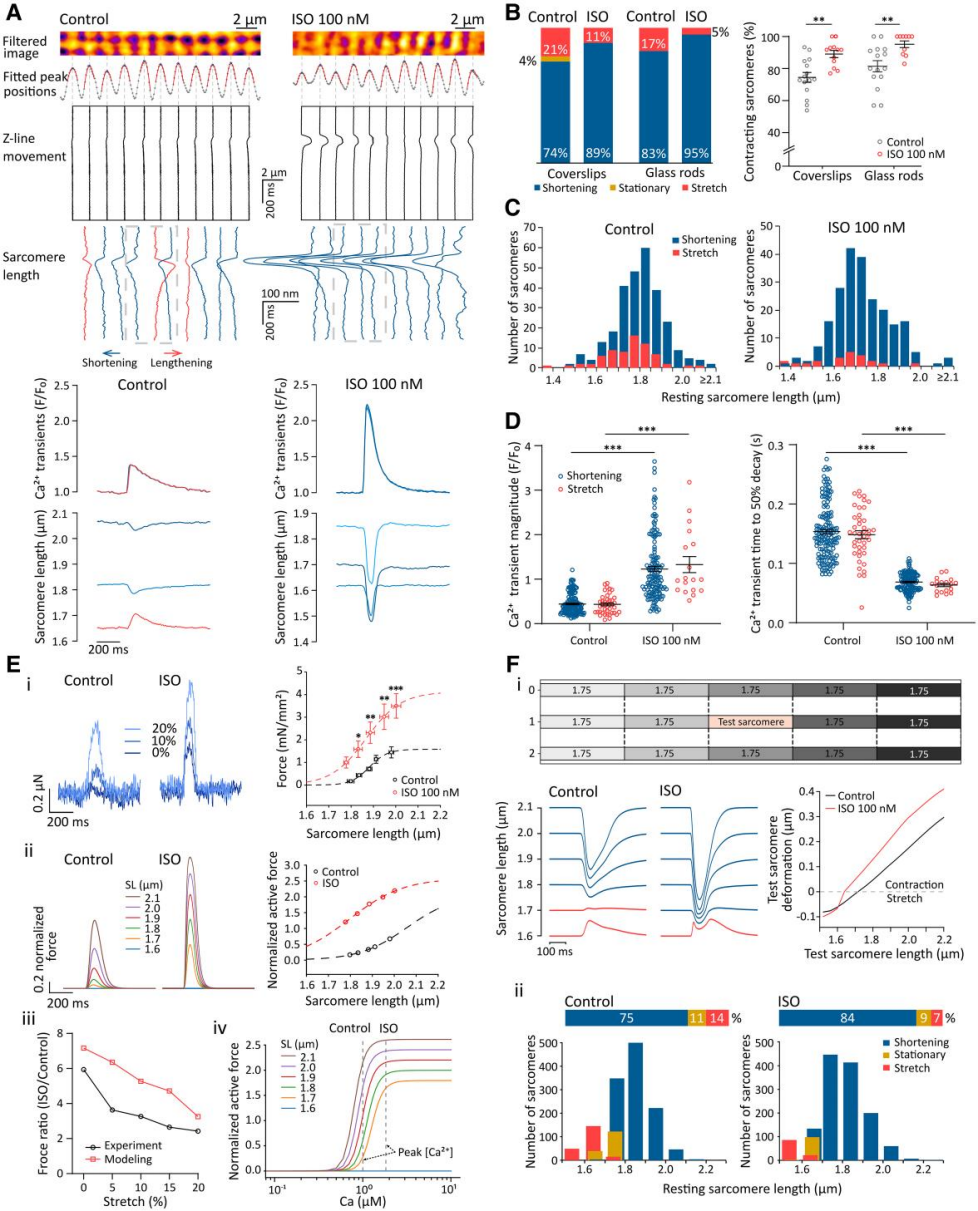
β-Adrenergic stimulation recruits additional contracting sarcomeres. (*A*) Z-line positions (brightfield imaging) and cytosolic Ca^2+^ (fluo-4) were simultaneously recorded using a high-speed camera.^1^ Extracted sarcomere deformations and corresponding local Ca^2+^ transients were compared ±100 nM ISO. Enlargements of recordings from neighbouring sarcomeres are shown in the lower panels. (*B*) Proportions of shortening, stretched, and stationary sarcomeres, for cells plated on coverslips or attached to glass rods. Corresponding SLs (*C*) and local Ca^2+^ transients (*D*). *n*_sarcomeres_, *n*_cells_, *n*_hearts_: plated cells control = 202, 15, 3; ISO = 162, 12, 3; glass rods control = 151, 15, 7; ISO = 82, 10, 6. (*E*) i, Representative and mean force recordings during step-wise cell stretch. *n*_cells_, *n*_hearts_: control = 8, 5; ISO = 8, 5. ii, Corresponding changes in the modelled force–length relationship. iii, Developed force ratio (ISO/control) from experimental and modelling data. iv, Modelled force–[Ca^2+^] relationship at different SLs. (*F*) i, Modelling with a 15-sarcomere array, with SL changed for the central sarcomere.^1^ ii, Modelling of a larger, 30 × 50 sarcomere array including experimental SL distributions. In both models, effects of simulated ISO were tested on the direction of sarcomere strain. Statistical analysis was performed by linear mixed models (*B*, plated cells) or two-way ANOVA repeat measurements (*E*i) for normally distributed data. Nonparametric tests included Mann–Whitney *U* test (*B*, attached cells) and Kruskal–Wallis test with Dunn’s correction (*D*) as in Li *et al.*^1^ (*P < 0.05; **P < 0.01; ***P < 0.001).

Animal procedures were approved by the Norwegian Animal Research Authority, in compliance with National Institutes of Health guidelines. Adult male C57BL/6 mice were anaesthetized by inhalation of 5% isoflurane/95% O_2_, and hearts were then rapidly excised for cannulation and isolation of cardiomyocytes.^1^ Transmitted light Z-line signals were used to assess changes in sarcomere length (SL)^1^ (*Figure 1A*). In contracting cells placed on coverslips (HEPES Tyrode’s solution, extracellular [Ca^2+^] = 1.0 mM, 37°C, 1 Hz), non-uniform sarcomere deformation was observed; although most sarcomeres shortened, a minority (≈20%) were lengthened or remained stationary (≈5%) (*Figure 1A* and *B*). During treatment with isoproterenol (ISO), overall contraction increased and sarcomere strain became more uniform, as more sarcomeres shortened (89 ± 2%).

To ensure that these observations did not reflect uneven cellular adherence to the coverslip, we also examined cardiomyocytes affixed to glass rods.^1^ Again, we observed non-uniform strain in untreated cells, with a significant proportion of stretched sarcomeres, but nearly complete recruitment of contracting sarcomeres during ISO (*Figure 1B*).

Careful examination of sarcomere strain recordings revealed a critical role of resting SL. Under control conditions, stretched sarcomeres exhibited shorter SL than those that contracted, as indicated by distribution plots (*Figure 1C*) and mean data (contracting SL = 1.80 ± 0.01 µm; stretched = 1.75 ± 0.01 µm, *P* < 0.05). Interestingly, it was predominantly these shorter sarcomeres that were recruited to contract during ISO treatment.

We investigated whether this finding is explained by differences in local Ca^2+^ transients within sarcomeres. As expected, ISO treatment augmented Ca^2+^ transients and accelerated Ca^2+^ decline (*Figure 1A*, lower panel). However, in both control and ISO, local Ca^2+^ transients were remarkably similar in contracting and stretched sarcomeres (*Figure 1D*), suggesting that differential Ca^2+^ delivery is unlikely to underlie strain inhomogeneity.

Greater force production by longer sarcomeres may allow their contraction at the expense of their shorter neighbours.^1^ But why would this relationship be altered during ISO treatment? We identified critical changes in the force–length relationship. Stepwise cell lengthening under control conditions produced a stereotypical force–length relationship (*Figure 1E*i). During ISO, force development was markedly augmented, as expected.^3^ However, force production was particularly large at short SLs, as the force–length relationship was shifted upwards.

We investigated whether this effect could allow short sarcomeres to contract during β-stimulation, using two mathematical models of sarcomere interactions^1^ derived from the Rice model.^4^ β-Stimulation was modelled by incorporating the observed increase in the Ca^2+^ transient (ISO:control ratio = 1.824), accelerated cross-bridge cycling (fits of force decline, ratio = 1.168), and reduced myofilament Ca^2+^ sensitivity (ratio = 0.8).^3,5^ Consistent with experiments, these changes yielded a disproportionately larger increase in force at short SLs (*Figure 1E*ii and iii). This is due to the sigmoidal shape of the force–Ca^2+^ relationship, which plateaus as all troponin C binding sites are occupied by Ca^2+^. Short sarcomeres begin at a much lower point on this curve, whereas longer sarcomeres operate closer to saturation. Consequently, during ISO, the larger Ca^2+^ transient allows short sarcomeres to achieve a much greater relative increase in force (*Figure 1E*iv).

To examine the functional outcome of these effects, we employed a dynamic model consisting of 15 sarcomeres and varied the length of the central test sarcomere (*Figure 1F*i**)**. Under control conditions, a shorter-than-average test sarcomere was stretched by its neighbours, but at longer lengths, it contracted. However, under simulated ISO conditions, the test sarcomere contracted even at short lengths (*Figure 1F*i).

We next employed a larger array of 30 by 50 sarcomeres,^1^ to include SL variability mirroring that observed in cells. These conditions reproduced experimental proportions of shortening and stretched sarcomeres, with β-stimulation recruiting more short sarcomeres to contract (*Figure 1F*ii).

In summary, β-adrenergic stimulation harmonizes sarcomere strain, as a large increase in force production by short sarcomeres allows them to contract rather than be stretched by their longer neighbours. Thus, increased sarcomere contractile recruitment may be an unrealized contributor to the fight-or-flight response. *In vivo* confirmation of this finding is expected to be hampered by motion artefacts in the beating heart, particularly during β-stimulation, which obfuscate single-sarcomere tracking. However, diastolic SL in the mouse heart is ≈1.70–1.90 µm;^2,6^ a range where we expect sarcomere recruitment to be dependent on β-adrenergic tone. Such recruitment may be impaired in disease states, particularly if titin expression/isoforms are altered, since this protein critically regulates SL variability.^1^ This topic will be the subject of future work.
